# Associations of Electrocardiographic Parameters with Left Ventricular Longitudinal Strain and Prognosis in Cardiac Light Chain Amyloidosis

**DOI:** 10.1038/s41598-019-44245-9

**Published:** 2019-05-23

**Authors:** Darae Kim, Ga Yeon Lee, Jin-Oh Choi, Kihyun Kim, Seok Jin Kim, Eun-Seok Jeon

**Affiliations:** 1 Division of Cardiology, Department of Medicine, Samsung Medical Center, Sungkyunkwan University School of Medicine, Seoul, Republic of Korea; 2 Divsion of Hemato-oncology, Department of Medicine, Samsung Medical Center, Sungkyunkwan University School of Medicine, Seoul, Republic of Korea

**Keywords:** Cardiomyopathies, Cardiovascular diseases

## Abstract

A 12-lead ECG is a simple and less costly measure to assess cardiac amyloidosis and may reflect the infiltrative nature of cardiac amyloidosis and have prognostic value for predicting overall survival in patients with cardiac AL amyloidosis. Therefore, we investigated the associations of surface ECG parameters with left ventricular (LV) global longitudinal strain (GLS) and prognosis in patients with cardiac AL amyloidosis. We performed a multi-center, retrospective analysis of 102 biopsy-proven cardiac AL amyloidosis patients. Baseline studies included 12-lead surface ECG and echocardiography, with two-dimensional strain analysis performed within one month of diagnosis. From the Kaplan-Meier survival analysis, patients with prolonged QTc (≥483 msec) had significantly poorer survival. ECG scores were assigned according to presence of prolonged QTc (≥483 msec) and abnormal QRS axis, and the study participants were divided into three groups according to ECG score. Mean absolute value of LV GLS and regional LV longitudinal strain (LS) differed significantly among the three groups and decreased in a stepwise manner as ECG score increased. Log NT-proBNP increased in a stepwise manner as ECG score increased. Prolonged QTc (≥483 msec) and abnormal QRS axis showed significant incremental values in addition to the revised Mayo stage. The presence of prolonged QTc (≥483 msec) and abnormal QRS axis showed significant incremental values for overall mortality rates. In addition, ECG scores consisting of presence of prolonged QTc (≥483 msec), and abnormal QRS axis showed good association with longitudinal LV dysfunction and NT-proBNP. ECG finding may provide prognostic additional information regarding prognosis of AL amyloidosis with cardiac involvement.

## Introduction

Primary light-chain (AL) amyloidosis is a plasma cell disorder marked by the deposition of immunoglobulin-derived amyloid in multiple organs, including the heart. Cardiac involvement is characterized by infiltrative cardiomyopathy, and it is the major determinant of overall survival in light-chain (AL) amyloidosis patients. Feasibility of intensive treatment and quality of life are often affected by cardiac involvement^1^. Therefore, assessing cardiac burden of amyloidosis is important for prognosis and future treatment strategy. Previous studies suggested that left ventricular (LV) global longitudinal strain (GLS), measured from two dimensional echocardiography with strain analysis has prognostic value in patients with AL amyloidosis and correlate with amyloid burdens^2–4^.

A 12-lead ECG is a simple and less costly measure to assess cardiac amyloidosis. We hypothesized that the features of a surface 12-lead ECG would reflect the infiltrative nature of cardiac amyloidosis and have prognostic value for predicting overall survival in patients with cardiac AL amyloidosis. Therefore, we investigated the features of surface ECG for association with prognosis for overall survival in cardiac AL amyloidosis. Also, we explored association of ECG parameters with LV GLS and regional longitudinal strains (LS).

## Results

### Baseline characteristics

During the median follow-up of 23 months, a total of 43 patients died. The mean age of all study participants was 61.6 years, and 63% were men. At baseline, a total of 48 patients (47%) were classified as revised Mayo Stage IV, and all other patients were at Stage III (Table 1). Low voltage in limb leads was the most prevalent abnormal finding (59%), followed by poor R progression (53%) and pseudoinfarction pattern (47%). The mean absolute value of LV GLS was 10 ± 4.2%. Regional LV LS showed a relative apical sparing pattern, and basal LV LS showed the lowest absolute value.

**Table 1:** Baseline characteristics of all patients.

	N = 102
Age (years)	61.6 ± 10.8
Men, n (%)	64 (63)
Body surface area (m^2^)	1.66 ± 0.19
**New York Heart Association class, n (%)**	
I	20 (19)
II	64 (63)
III	15 (15)
IV	3 (3)
Systolic blood pressure (mmHg)	102.0 ± 15.7
Diastolic blood pressure (mmHg)	64.7 ± 9.4
Log NT-proBNP (pg/mL)	3.60 ± 0.49
Troponin T (ng/mL)	0.08 ± 0.05
Creatinine (g/dL)	1.26 ± 1.33
eGFR (mL/min/1.73 m2)	75.7 ± 31.8
Revised Mayo stage IV, n (%)	48 (47)
Autologous stem cell transplantation, n (%)	15 (15)
Chemotherapy, n (%)	83 (81)
**Electrocardiographic parameters**	
PR interval (msec)	180.0 ± 31.5
Pseudoinfarction pattern, n (%)	48 (47)
Poor R wave progression, n (%)	54 (53)
**Low voltage, n (%)**	
Limb leads	60 (59)
Precordial	5 (5)
Sokolow index (mm)	6.1 ± 4.2
LV hypertrophy pattern, n (%)	10 (10)
Fragmented QRS, n (%)	14 (14)
QRS axis (°)	37.0 ± 98.6
QRS duration (msec)	99.4 ± 27.5
QTc(msec)	466.7 ± 37.5
**Echocardiographic parameters**	
LV end diastolic dimension, mm	45.6 ± 5.2
LV end systolic dimension, mm	30.6 ± 5.0
**LV thickness, mm**	
Interventricular septum	13.6 ± 2.6
Posterior wall	12.7 ± 2.2
Relative wall thickness	0.58 ± 0.14
LV mass index (g/m^2^)	143.4 ± 35.9.
LV ejection fraction (%)	55.1 ± 9.5
LA volume index (ml/m^2^)	50.2 ± 19.8
Septal E’ velocity (cm/s)	0.04 ± 0.01
E/E’	22.7 ± 12.1
LV GLS, %	−10.2 ± 4.2
**Regional LV LS, %**	
Basal	−7.35 ± 3.80
Mid	−9.50 ± 4.29
Apex	−14.0 ± 6.18

Median (range), mean ± SD, eGFR: estimated glomerular filtration rate.

LV, left ventricle; LA, left atrium; GLS, global longitudinal strain; LS, longitudinal strain.

### Prognostic values of electrographic parameters

**Figure 1:**
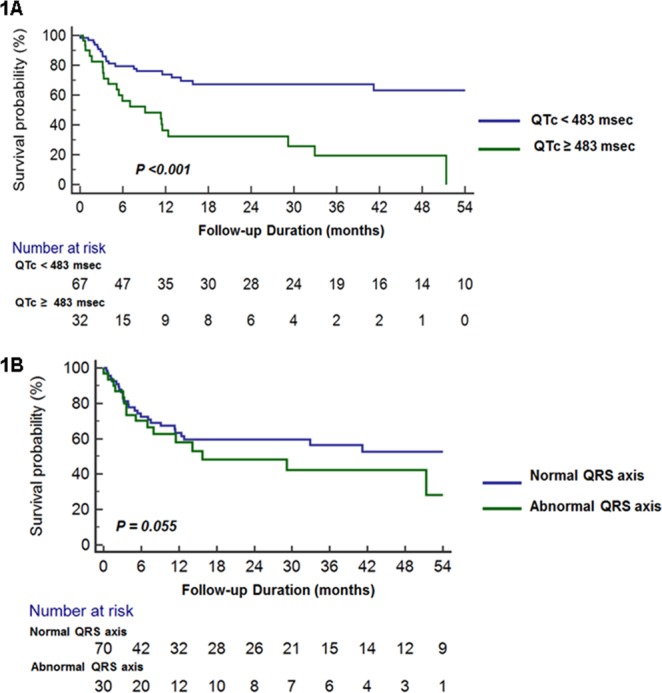
(**A**) Kaplan-Meier survival curves according to presence of prolonged QTc (≥483 msec). Patients with prolonged QTc at baseline ECG had significantly worse overall survival rates. (**B**) Kaplan-Meier survival curves according to presence of abnormal QRS axis. Patients with abnormal QRS axis at baseline ECG showed a trend of poor overall survival, although the p value was not significant (p = 0.055).

The QTc interval showed satisfactory predictive values for overall survival in cardiac AL amyloidosis (Supplementary Fig. 1, cut-off value: 483 msec, sensitivity 53%, specificity 80%, AUC 0.652 [95% CI: 0.536–0.768], p = 0.012). Among the electrocardiographic parameters, prolonged QTc (≥483 msec) (p = 0.002, 95% confidence interval: 1.562–9.221), and abnormal QRS axis (p = 0.049, 95% confidence interval: 1.004–5.435) showed significant association with overall mortality from univariate analysis (Table 2). Patients with prolonged QTc (≥483 msec) had significantly poorer overall survival rates compared to those without prolonged QTc (<483 msec) at baseline ECG (Fig. 1A). Patients with abnormal QRS axis showed a trend of worse overall survival rates; however, the p value was not significant (p = 0.055, Fig. 1B).

**Table 2:** Univariate and multivariate analyses for overall mortality.

	Univariate analysis		Multivariate analysis	
	HR (95% CI)	P value	HR (95% CI)	P value
Age, years	0.963–1.039	0.989		
Male gender	0.442–2.247	0.994		
eGFR (mL/min/1.73 m^2^)	0.986–1.015	0.932		
Revised Mayo stage IV	1.592–8.322	0.002	0.589–2.645	0.564
Abnormal QRS axis	1.004–5.435	0.049	1.303–4.724	0.131
Prolonged QTc (≥483 msec)	1.562–9.221	0.003	1.627–2.666	0.006
Poor R progression	0.494–2.419	0.826		
Prolonged PR	0.321–1.977	0.624		
Low voltage (limb leads), mm	0.505–2.503	0.774		
Low voltage (Precordial leads), mm	0.036–3.114	0.337		
Sokolow index ≤1.5 mV	0.287–2.605	0.717		
Pseudo-infarct pattern	0.456–2.223	1.006		
Fragmented QRS	0.331–3.232	0.954		
LV hypertrophy pattern	0.245–3.523	0.915		
QRS duration	0.987–1.015	0.909		
Mean LV wall thickness	0.974–1.269	0.118		
LV GLS	1.067–1.258	<0.001	1.004–1.221	0.041

LV. left ventricle; GLS, global longitudinal strain.

### Correlation between ECG scores and LV function, NT-proBNP, and prognosis

**Figure 2:**
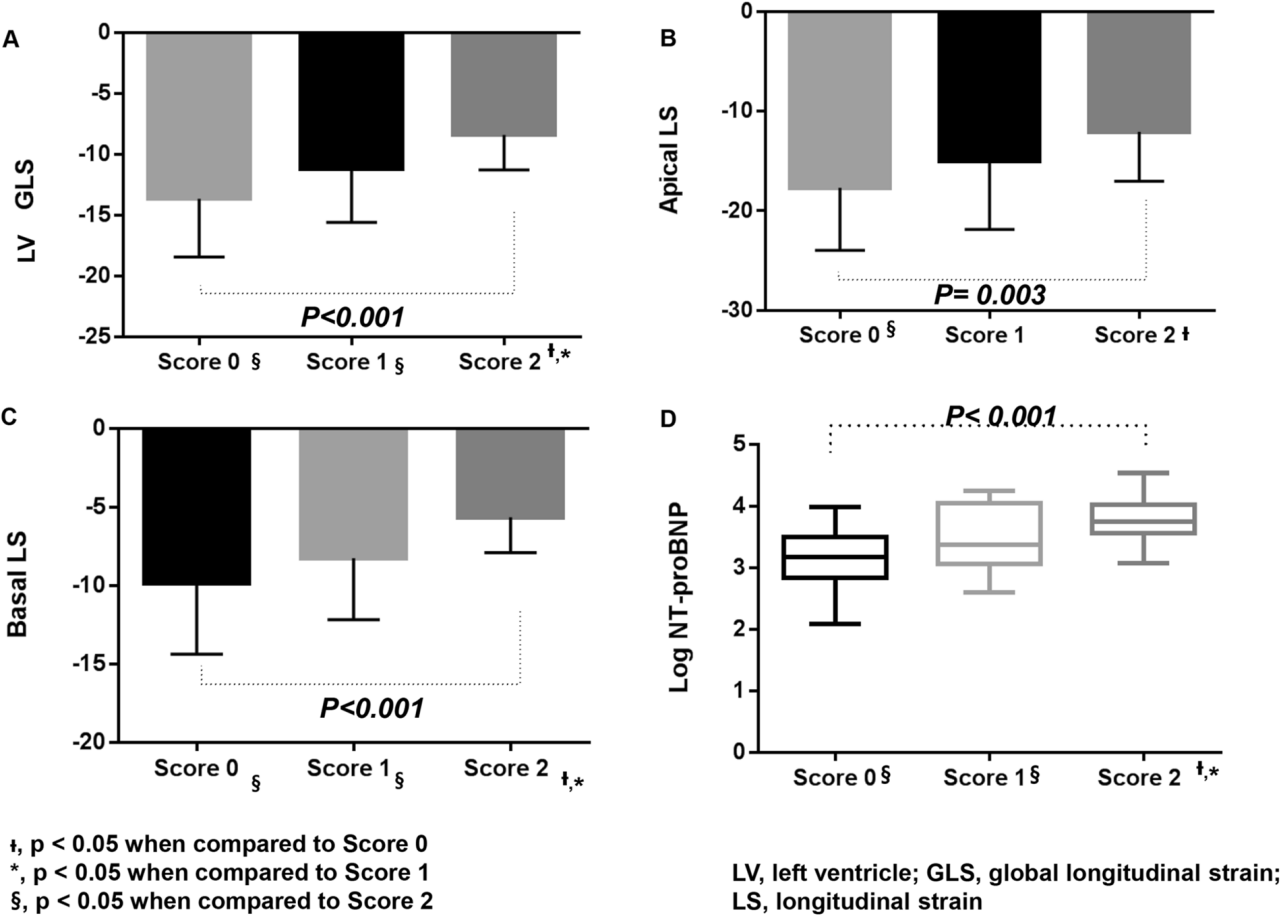
Patient ECGs were assigned 1 point for each presence of prolonged QTc (≥483 msec) and abnormal QRS axis. Patients were classified into three groups according to ECG score. The mean values of LV GLS (**A**) apical LV LS (**B**) and basal LV LS (**C**) differed significantly among the three groups. The mean values of log NT-proBNP (**D**) also differed significantly among the three groups.

The patient’s ECGs were scored by assigning 1 point for the presence of a prolonged QTc (≥483 msec) or abnormal QRS axis (ECG scores). The patients were classified into three groups according to ECG scores: patients with neither prolonged QTc (≥483 msec) nor abnormal QRS axis (score 0, n = 15), patients with either prolonged QTc (≥483 msec) or abnormal QRS axis (score 1, n = 39), and patients with both prolonged QTc (≥483 msec) and abnormal QRS axis (score 2, n = 48). The mean values of LV GLS, basal LV LS, and apical LV LS differed significantly among the three groups (Fig. 2A–C), and these values decreased in a stepwise manner as the ECG score increased. The mean values of log NT-proBNP differed significantly among the three groups (p < 0.001), and these values increased in a stepwise manner as the ECG score increased (Fig. 2D).

Prolonged QTc (≥483 msec) and abnormal QRS axis showed significant incremental values for overall survival in addition to revised Mayo stage (Supplementary Fig. 2). From multivariate modeling, prolonged QTc (≥483 msec) was the only independent electrocardiographic parameter for overall mortality when adjusted by revised Mayo Stage and LV GLS (Table 2).

## Discussion

In the present study, we evaluated the associations of surface ECG parameters between LV GLS and prognosis of AL amyloidosis patients with cardiac involvement. To the best of our knowledge, this is the first study to evaluate the prognostic values of prolonged QTc and abnormal QRS axis in patients with cardiac AL amyloidosis proven by endomyocardial biopsy. We report the following principle findings in patients with cardiac AL amyloidosis: (1) Prolonged QTc (≥483 msec) and abnormal QRS axis showed significant incremental prognostic values in addition to revised Mayo stage; (2) ECG scores which were assigned by presence of prolonged QTc (≥483 msec) and abnormal QRS axis, showed good associations with the mean absolute values of LV GLS and regional LS; and (3) mean values of Log NT-proBNP differed significantly according to ECG score and showed an increasing trend as ECG score increased.

The ECG is a simple and low cost diagnostic evaluation. The diagnostic value of surface ECG for cardiac amyloidosis is well known^5,6^. However, only a few studies have investigated the prognostic values of surface ECG parameters in the setting of biopsy-proven cardiac amyloidosis. In this study, we hypothesized that surface ECG may reflect the amyloid burden in cardiac AL amyloidosis and, therefore, may associate with LV GLS and prognosis.

Similar to previous studies, the present investigation showed that low voltage in limb leads (59%), poor R progression (53%), and pseudoinfarction (47%) were the most common findings^7^. However, they were not associated with overall survival or LV GLS. A previous study by Cyrille *et al*.^8^ suggested that low voltage QRS, defined as Sokolow index ≤1.5 mV, was independently associated with adverse outcomes in cardiac amyloidosis^8^. In our study, neither Sokolow index ≤1.5 mV nor low voltage QRS (either at limb leads or precordial leads) was associated with overall survival. This difference may result from different study populations. The study by Cyrille *et al*.^8^ included both AL and transthyretin amyloidosis, which have different prognoses and disease profiles^1^. In addition, they included patients with image-based diagnosis of cardiac involvement if the histological documentation of Congo red staining was positive from other organs, which could have missed early involvement of cardiac amyloidosis. As they mentioned in their study, low voltage pattern could be less prevalent in early stages of cardiac amyloidosis. In our study, we included only patients with cardiac AL amyloidosis confirmed by endocardial biopsy, a confirmatory diagnostic method, and there were some patients with equivocal echocardiographic findings even with positive endocardial biopsy. Previous studies have suggested that the presence of pseudoinfarction and fragmented QRS is associated with prognosis in cardiac AL amyloidosis defined by either endomyocardial biopsy or echocardiographic evidence in systemic amyloidosis^9,10^. However, neither pseudoinfarction nor fragmented QRS showed prognostic value in our study.

A prolonged QTc suggests repolarization abnormality. We speculate that QTc prolongation represents repolarization heterogeneity in cardiac AL amyloidosis, which may be associated with the infiltrative nature of the disease and may reflect amyloid burden. The QTc interval in our study population showed modest correlation with LV GLS (r = 0.336, p = 0.001), suggesting an association between LV longitudinal mechanical dysfunction and electrocardiographic repolarization abnormality (Supplementary Fig. 3A). The prognostic value of the QTc interval has been suggested in healthy populations and those with cardiovascular diseases^11–15^. From our results, prolonged QTc interval remained independently associated with overall mortality, even after adjusting for revised Mayo stage, LV GLS, and other variables. In addition, the QTc interval showed a modest correlation with log NT-proBNP (r = 0.406, p < 0.001) (Supplementary Fig. 3B). An abnormal QRS axis also showed significant incremental values in addition to revised Mayo stage and prolonged QTc interval. An abnormal QRS axis may indicate infiltrative burden precluding a normal electrical conduction vector.

We suggested ECG scores consisting of prolonged QTc interval and abnormal QRS axis. LV longitudinal function showed a decreasing trend as scores increased, while log NT-proBNP showed an increasing trend as scored decreased. Our results suggest that ECG scores may reflect amyloid burdens in cardiac amyloidosis. Supplementary Table 1 shows the comparisons of baseline characteristics among patients when classified according to ECG score. Baseline clinical characteristics were similar among three groups. Mean LV wall thickness and LV ejection fraction were similar among three groups, but LV end systolic dimensions increased significantly as ECG scores increased. ECG scores were associated with some of ECG parameters. The presence of low voltage QRS in limb leads and pseudoinfarction pattern differed significantly among the three groups, and their prevalence increased as ECG score increased.

## Limitations

The present study has several limitations that need to be addressed. First, treatment response is an important predictor of survival in AL amyloidosis, but this was not included because evaluation of response status was not achievable due to limited survival of patients in our cohorts. When subgroup analysis was performed according to chemotherapy regimen (Supplementary Table 1), there was no significant difference of overall survival according to chemotherapy regimen (Supplementary Fig. 4). Secondly, we did not include cardiac AL amyloidosis patients with pacemaker implantation or persistent atrial fibrillation. These patients could not be included because GLS is variable in this population subgroup, and this may have induced a selection bias in the population. Thirdly, we did not include more sophisticated QT-related electrocardiographic measures, such as QT dispersion or T peak-T end. The goal of the current study, however, was to assess the prognostic value of simple, readily available, 12-lead surfaces, such as the QTc interval.

In conclusion, the presence of prolonged QTc (≥483 msec) and abnormal QRS axis showed significant incremental value for overall mortality rate in AL amyloidosis patients with cardiac involvement. ECG scores consist of presence of prolonged QTc (≥483 msec), and abnormal QRS axis showed good association with longitudinal LV dysfunction and cardiac biomarker, NT-proBNP. Surface ECG findings may provide prognostic additional information regarding prognosis of AL amyloidosis with cardiac involvement.

## Methods

### Study population

We retrospectively reviewed the medical records of patients who were diagnosed with systemic amyloidosis from January 2009 to December 2016, at Seoul National University Hospital and Samsung Medical Center. All patients had biopsy-proven amyloidosis confirmed by Congo red staining and immunohistochemistry of any tissue specimen. Cardiac involvement was confirmed by endomyocardial biopsy, and these patients were included in the study. Any patient with poor echocardiographic images, no follow-up information, persistent atrial fibrillation, paced rhythm, or previous history of myocardial infarction was excluded. In total, 102 patients with cardiac AL amyloidosis were analyzed (n = 69 from Samsung Medical Center, n = 33 from Seoul National University Hospital).

The baseline clinical information, laboratory results from blood samples, 12-lead electrocardiography (ECG), and two-dimensional transthoracic echocardiography at the time of diagnosis were reviewed. The Modification of Diet in Renal Disease (MDRD) equation was used to calculate estimated glomerular filtration rate (eGFR).

The mortality endpoint was defined as the time to all-cause death from baseline for all deceased patients and time to censor date (November 30^th^, 2017) for all other patients. Date of baseline was within one month of diagnosis. Occurrence and date of death and ongoing survival status were routinely monitored by regular visits or telephone calls. The median follow-up duration of the censored cases was 23.0 ± 26.5 months.

### 12-lead ECG

Analysis of the baseline standard 12-lead electrocardiogram was performed by two independent readers who were blinded to echocardiographic data, cardiac biomarkers, and clinical data. Baseline ECG was performed within one month of diagnosis.

The baseline 12-lead ECG was analyzed for the following characteristics: rhythm, prolonged PR (>200 msec) interval, low voltage (defined by amplitude of QRS in each limb lead ≤0.5 mV^1,16–18^ or precordial lead ≤1 mV^5^), and pseudo-infarct pattern (defined by pathologic Q waves or QS waves in at last two consecutive leads in the absence of myocardial infarction and left bundle branch block). QRS voltage amplitude was measured as the sum of the S wave in V1 and the R wave in V5 or V6 and presented as the Sokolow index^8^. LV hypertrophy was defined by the Sokolow-Lyon’s criteria^19^ (S wave in V1 and R wave in V5 or V6 ≥ 3.5 mV) and by the Cornell criteria (R wave in aVL and S wave in V3 > 2.8 mV in men or >2 mV in women or R wave in aVL > 1.2 mV)^20^. Patients with left bundle branch block on electrocardiogram were excluded from the analysis of left ventricular hypertrophy and pseudoinfarct^8^. Poor R progression was determined if the R wave in V3 was ≤3 mm. Fragmented QRS was defined by the presence of various RSR’ patterns with or without a Q wave and included an additional R wave (R), notching of the R wave, notching of the downstroke or upstroke of the S wave, and the presence of >1 R′ in two contiguous leads corresponding to a major coronary artery territory^9^. Normal QRS axis was defined as ≥−30° and <90°. The QRS, QT, QT corrected interval (QTc), and PR interval were measured in all patients.

### Echocardiography

All patients underwent standard baseline echocardiography according to the previous guidelines using Vivid 7 or Vivid 9 cardiovascular ultrasound system (GE Medical Systems, Horten, Norway)^21^. Left ventricular (LV) end diastolic dimension, end systolic dimension, interventricular septal thickness, LV posterior wall thickness, and left atrial volume index were measured. Mean LV wall thickness was calculated by averaging the thickness of the interventricular septum and the posterior wall. The ratio of early transmitral flow to early septal mitral annular diastolic velocity (E/e’) was measured as an index of LV filling pressure.

For two-dimensional strain analysis, three consecutive cardiac cycles were recorded and averaged, and the frame rates were set to 60–80 frames/sec. The analysis was performed offline using customized software (EchoPAC PC, version 113; GE Medical Systems). The endocardial border of the LV was manually traced from three apical views (apical 4-, 2-, and 3-chamber views) to obtain LV GLS. In addition, regional LV longitudinal strain (LS) values were calculated by averaging six basal, six mid, and six apical segments to obtain basal, mid, and apical LV LS^22,23^.

### Statistical analysis

Continuous data were expressed as mean ± standard deviation, and categorical variables were expressed as absolute number (percent). Receiver operating characteristic (ROC) curves were used to assess the predictive accuracy of QTc on overall mortality. Differences in overall survival according to QTc or QRS axis were assessed using log-rank analysis with right censoring and displayed by Kaplan-Meier survival curves. Patients were scored according to presence of QTc ≥483 msec and abnormal QRS axis. Continuous variables were compared among the three groups using one-way ANOVA. Univariate and multivariate Cox proportional hazard analyses were conducted to evaluate associations between baseline variables (clinical and ECG) and overall survival. The incremental prognostic utility of ECG parameters was assessed by comparing the global chi-square values. All analyses were performed using SPSS version 24 (SPSS Inc., Chicago, Ill., USA). A p value < 0.05 was considered statistically significant.

### Ethical considerations

This study was approved by the Ethics committee of Samsung Medical Center. Informed consent was obtained from all subjects, and all methods were carried out in accordance with the relevant guidelines and regulations according to the principles expressed in the Declaration of Helsinki. All study protocols were approved by Institutional Review Board at Samsung Medical Center.

## Supplementary information

supplementary tables and figures

## Data Availability

The datasets generated during and/or analyzed during the current study are available from the corresponding author on reasonable request.
