# Supplementary material for: Associations of Electrocardiographic Parameters with Left Ventricular Longitudinal Strain and Prognosis in Cardiac Light Chain Amyloidosis
Source: Sci Rep. 2019 May 23;9:7746. doi: 10.1038/s41598-019-44245-9 (PMC6533364; doi:10.1038/s41598-019-44245-9)
Supplement: Supplementary file 1 — supplementary tables and figures [file 41598_2019_44245_MOESM1_ESM.pdf]

Associations of Electrocardiographic Parameters with Left Ventricular Longitudinal Strain and Prognosis in Cardiac Light Chain Amyloidosis

Darae Kim, MD, PhD[1], Ga Yeon Lee, MD[1], Jin-Oh Choi, MD, PhD[1], Kihyun Kim, MD, PhD[2], Seok Jin Kim MD, PhD[2], Eun-Seok Jeon, MD,PhD[1]

[1]Division of Cardiology, Department of Medicine, [2]Divsion of Hemato-oncology, Department of Medicine, Samsung Medical Center, Sungkyunkwan University School of Medicine, Seoul, Republic of Korea

Short titles: Prognostic values of ECG in AL amyloidosis

***Corresponding Author:** Eun-Seok Jeon, MD, PhD.

Division of Cardiology, Department of Medicine, Samsung Medical Center

81 Irwon-Ro Gangnam-gu, Seoul, Korea, 06351

Phone: +82-2-3410-3419, E-mail: eunseok.jeon@samsung.com

Supplementary Table 1. Comparisons of baseline characteristics according to chemotherapy regimen.

	BDex+AA (n=23)	Other regimen (n=28)	p-value
Men	15 (65)	16 (57)	0.383
Age	63.3±9.1	58.4±11.7	0.109
Revised Mayo stage IV, n (%)	16 (80)	16 (64)	0.200
ECG parameters			
PR interval (msec)	195.1 ± 29.2	171.6 ± 20.2	0.002
Pseudoinfarction pattern, n (%)	15 (68)	13 (46)	0.105
Poor R wave progression, n (%)	8 (36)	15 (53)	0.177
Low voltage, n (%)			
Limb leads	12 (52)	17 (60)	0.581
Precordial	0 (0)	1(4)	0.371
Sokolow index (mm)	7.8 ± 5.1	5.9 ± 4.4	0.167
LV hypertrophy pattern, n (%)	1 (5)	2 (7)	0.591
Fragmented QRS, n (%)	4(17)	3 (11)	0.387
QRS axis (°)	9.91 ± 116.2	62.2±84.3	0.079
QRS duration (msec)	108.1 ± 39.2	86.8±15.5	0.020
QTc(msec)	484.7 ± 33.8	447.6 ± 37.4	0.001
Echocardiographic parameters			
LVEDD, mm	45.4±4.5	44.7±5.8	0.609
LV ESD, mm	30.2±4.6	29.5±5.0	0.616
LV thickness, mm			
Interventricular septum	13.5±2.4	13.9±2.8	0.651
Posterior wall	12.6±2.2	13.2±2.5	0.333
Relative wall thickness	0.58±0.12	0.62±0.15	0.366
LV mass index (g/m^2)	140.0±30.2	146.5±44.2	0.549

LV ejection fraction (%)	53.2±11.1	57.1±9.1	0.185
LA volume index (ml/m^2)			
Septal E' velocity (cm/s)	0.03±0.01	0.04±0.01	0.019
E/E'	24.5 ± 14.6	19.9 ± 7.3	0.167
LV GLS, %	-7.9±3.5	-9.3±3.1	0.114
Regional LV LS, %			
Basal	-5.7 ± 2.6	-7.4±3.3	0.005
Mid	-7.3 ± 3.2	-9.0 ± 3.5	0.082
Apex	-10.8 ± 5.3	-11.5±3.8	0.584

BDex +AA, bortezomid-dexamethasone-cyclophoaphamide/melphalan regimen.

Other regimen includes high dose dexamethasone based regimens combined with melphalan, thalidomide, cyclophosphamide, and cyclophosphamide-thalidomide

Median (range), mean ± SD, eGFR: estimated glomerular filtration rate

LV, left ventricle; LA, left atrium; GLS, global longitudinal strain; LS, longitudinal strain

Supplement Figure 1. QTc interval at baseline ECG showed satisfactory predictive value for overall survival rate.

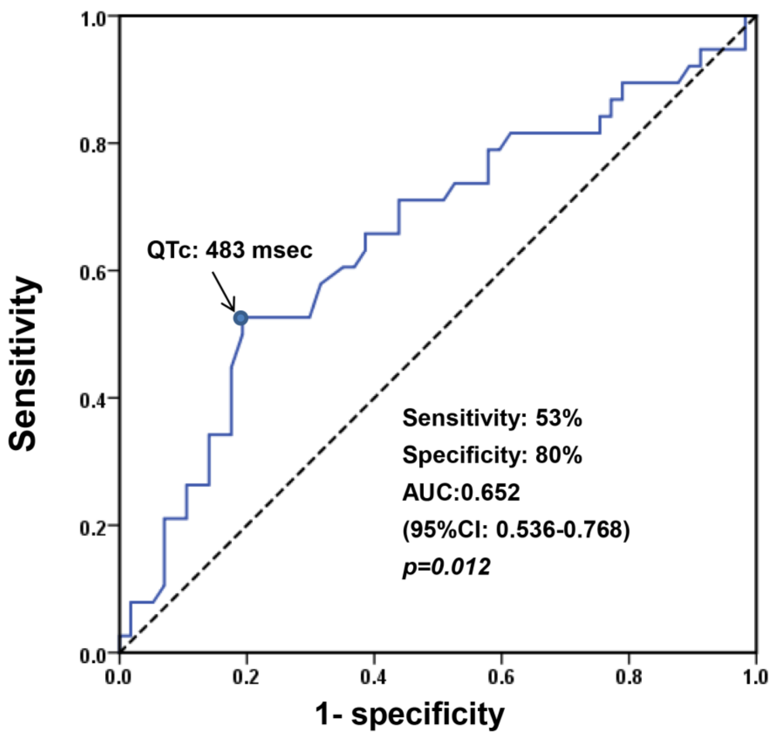

Supplement Figure 2. Prolonged QTc (≥ 483 msec) and abnormal QRS axis showed significant incremental value for overall survival in addition to revised Mayo stage in a stepwise manner.

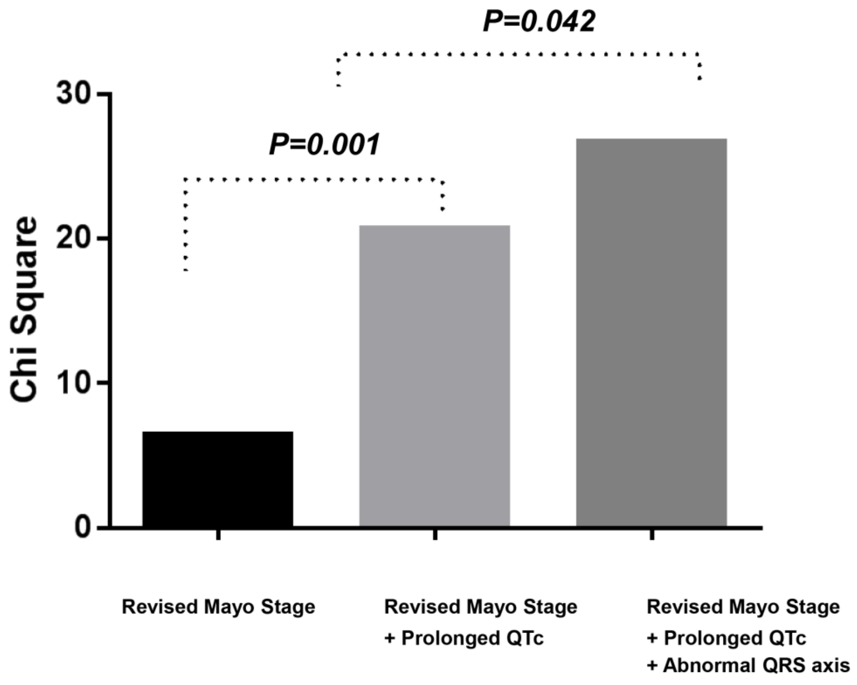

Supplement Figure 3A. Correlation between QTc interval and LV GLS, **3B**. Correlation between QTc interval and log NT-proBNP.

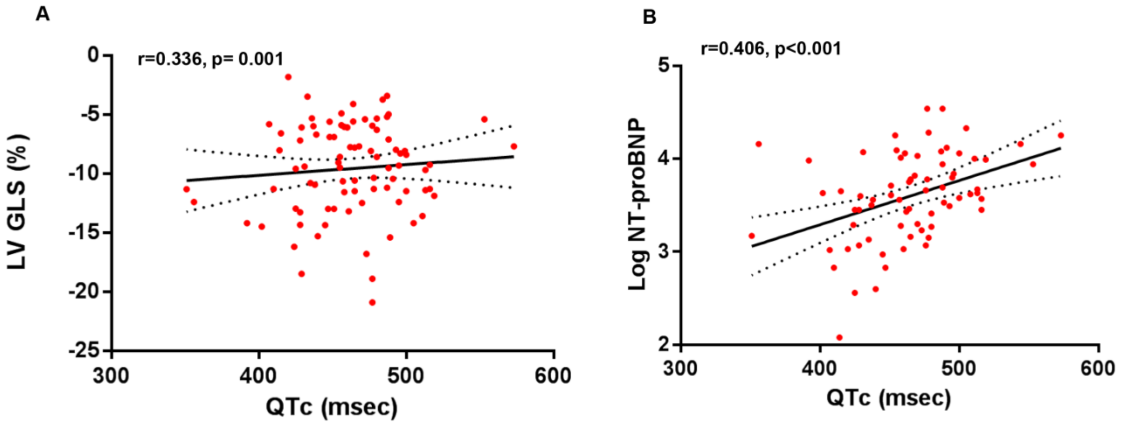

Supplement Figure 4. Kaplan-Meier survival curves according to chemotherapy regimen.

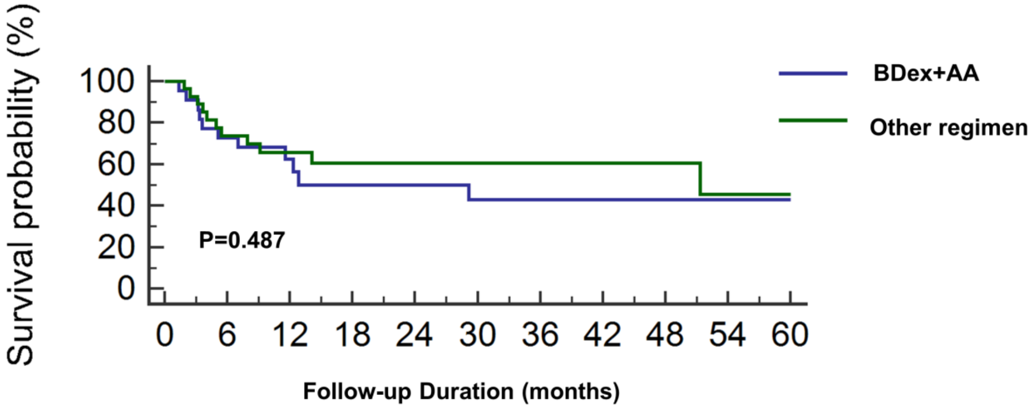

Number at risk

	0	6	12	18	24	30	36	42	48	54	60
BDex+AA	23	16	10	8	7	6	6	5	3	1	1
Other regimen	28	19	15	12	10	10	6	5	4	2	1

BDex +AA, bortezomid-dexamethasone-cyclophoaphamide/melphalan regimen.

Other regimen includes high dose dexamethasone based regimens combined with melphalan, thalidomide, cyclophosphamide, and cyclophosphamide-thalidomide
